# Use of an amplatzer vascular plug in embolization of a pulmonary artery aneurysm in a case of hughes-stovin syndrome: a case report

**DOI:** 10.1186/1752-1947-5-425

**Published:** 2011-09-01

**Authors:** Vasileios D Tzilalis, Georgios Vourliotakis, Ioannis A Tsironis, Vasileios D Tsiligiris, Elias N Brountzos

**Affiliations:** 1 Division of Vascular Surgery, Department of Surgery, 401 General Military Hospital of Athens, Kanelopoulou Street and Mesogion Avenue, 11525 Athens, Greece; 2 Second Department of Radiology, Eugenidion Hospital, Athens University Medical School, Athens, Greece

## Abstract

**Introduction:**

Hughes-Stovin syndrome is a rare condition characterized by peripheral deep venous thrombosis accompanied by single or multiple pulmonary arterial aneurysms. The limited number of cases has precluded controlled studies of the management of pulmonary artery aneurysms, which usually cause massive hemoptysis leading to death. This is the first report of a new endovascular treatment of a single large pulmonary arterial aneurysm.

**Case presentation:**

An 18-year-old Caucasian man was referred to our department with recurrent severe hemoptysis. His medical history included Hughes-Stovin syndrome diagnosed during a recent hospital admission. The patient was initially treated with corticosteroids. Because of his recurrent hemoptysis, we decided to embolize a 3.5 cm pulmonary arterial aneurysm using an Amplatzer Vascular Plug. The procedure was not complicated, and the patient's post-intervention course was uneventful. The patient has remained free from any complications of the embolization 36 months after the procedure.

**Conclusion:**

Percutaneous embolization of a single large pulmonary artery aneurysm with an Amplatzer Vascular Plug in a patient with Hughes-Stovin syndrome is a less invasive procedure that represents the best multidisciplinary approach in treating these patients.

## Introduction

Hughes-Stovin syndrome is a rare condition first described in 1959 that is characterized by peripheral deep venous thrombosis (DVT) often involving the vena cava and accompanied by single or multiple pulmonary arterial aneurysms [1]. The syndrome's typical symptoms are recurrent cough, dyspnea, chest pain, fever, and hemoptysis. The natural course of the disease is usually fatal because of fulminant hemoptysis. The patients with this syndrome are often men in the second to fourth decades of life. The pathogenesis of the syndrome remains obscure. In this report, we describe a new endovascular treatment of a single large pulmonary arterial aneurysm in a patient with Hughes-Stovin syndrome which, to our knowledge, is the first of its kind published in the literature.

## Case presentation

An 18-year-old Caucasian man with recurrent hemoptysis was referred to our hospital. A diagnosis of Hughes-Stovin syndrome had already been established in a previous hospital admission [2]. He had had some incidents of spontaneous non-productive cough several days prior to his admission.

**Figure 1:**
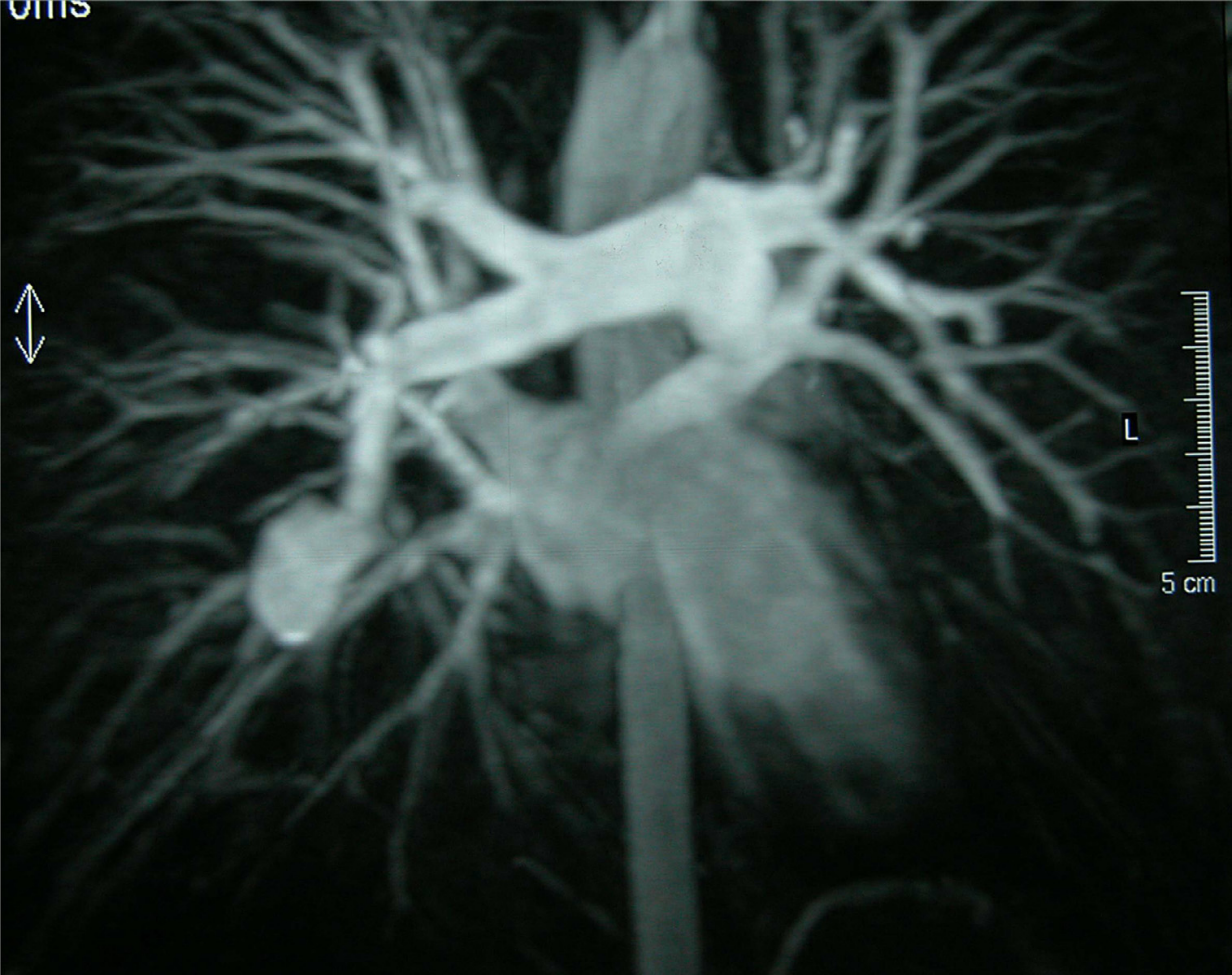
**Pre-operative MRA of the pulmonary arteries showing an aneurysm of the right lower lobe pulmonary artery with a diameter of 3.5 cm in the lower lobe of the right lung**.

During his previous admission three months before his current presentation, his physical examination was normal apart from mild, tender swelling of his left thigh and calf caused by DVT (left external iliac and femoral vein) detected by color duplex examination. His oropharynx and larynx were normal, and his mucous membranes were free of ulcers. Chest radiography revealed a round opacity in the right medial pulmonary field. CT of the chest showed an aneurysm in the right lower lobe pulmonary artery, together with mural thrombus, that had a diameter of 3.5 cm in the lower lobe of the right lung. Magnetic resonance angiography (MRA) of the cerebral venosus sinuses and the pulmonary arteries (Figure 1) was performed to clarify the etiology of the patient's headaches and to plan possible embolization of the aneurysm. Cerebral MRA revealed multiple chronic thromboses of the sclera mater venous sinuses. The rare diagnosis of systemic vasculitis Hughes-Stovin syndrome was made on the basis of the pulmonary artery aneurysm and peripheral venous thromboses in such a young patient. There were no findings consistent with Behçet's disease.

The patient was initially treated with corticosteroids. His hemoptysis resolved with intravenous methylprednisolone treatment after about 25 days. Upon his second admission, because of his recurrent hemoptysis, we decided to perform percutaneous embolization of the pulmonary artery aneurysm.

**Figure 2:**
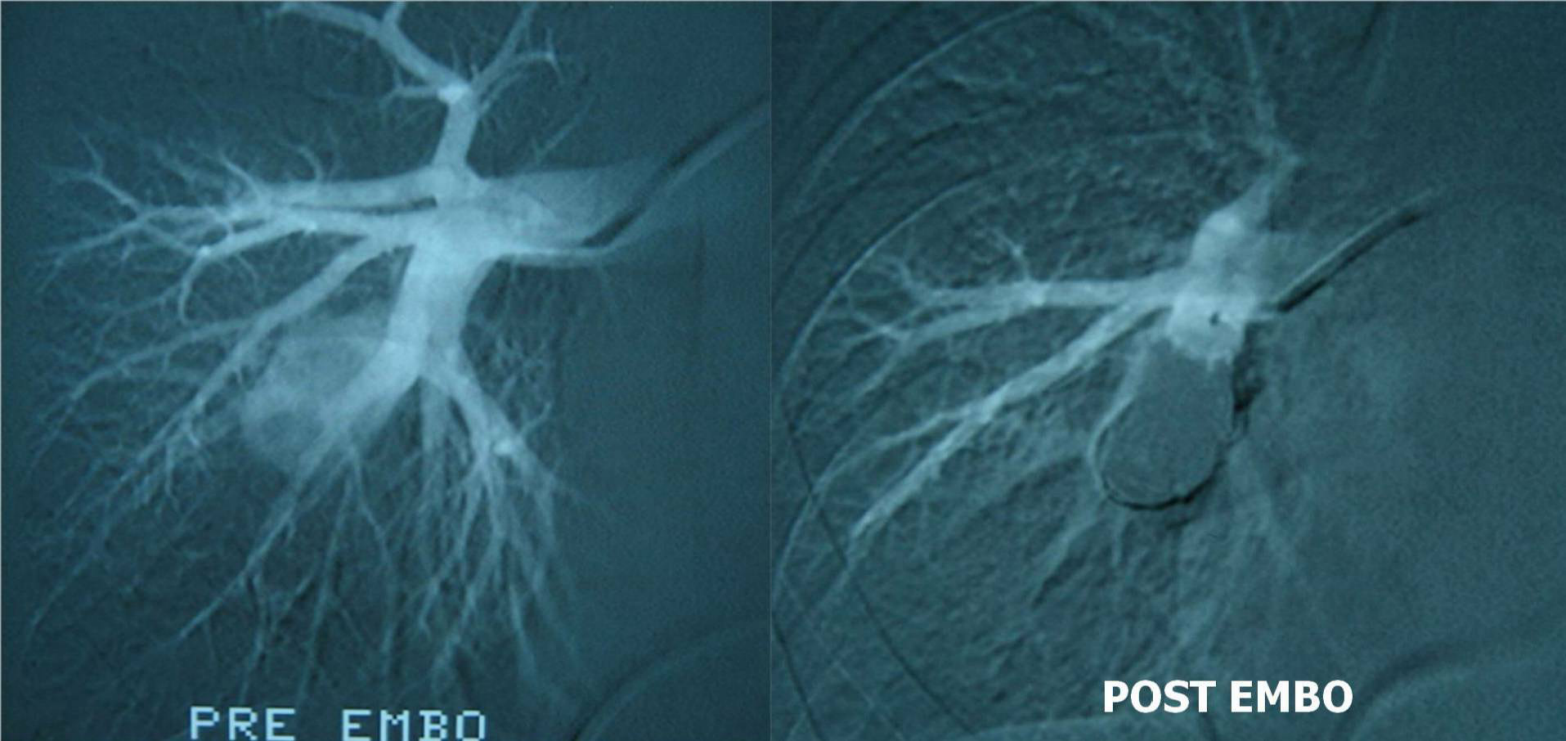
**Angiographic images demonstrating the successful embolization of the pulmonary artery aneurysm**.

The aneurysm was initially embolized with steel microcoils via an infusion catheter. Subsequently, an Amplatzer Vascular Plug (AGA Medical Corp., Plymouth, MN, USA) with a diameter of 16 mm was inserted into the cavity of the aneurysm. The procedure ended without any complications, and afterward there was no evidence of the pulmonary embolism (Figure 2). The patient's post-interventional course was uneventful, and he was discharged 30 days after the procedure. He was prescribed anti-coagulants (acenocoumarol) and immunosuppressant corticosteroids (methylprednisolone) to control his vasculitis. The patient developed a new DVT in his lower extremities during follow-up (in the 30th month) after temporary discontinuation of anti-coagulant treatment. Nevertheless, he has not had any complications from the aneurysm 36 months after undergoing embolization, and follow-up CT showed the completely thrombosed pulmonary artery aneurysm.

## Discussion

The limited number of patients with identified Hughes-Stovin syndrome has precluded the initiation of controlled studies of the management of patients with pulmonary arterial aneurysms [3]. Corticosteroids, alone or in combination with an immunosuppressant, are generally considered first-line therapy [4]. Surgical resection has been the traditional treatment of choice when the risk of lethal hemoptysis necessitates more definitive therapy. Total pneumonectomy or lobectomy has been performed with some successful results [5].

The high morbidity associated with surgery and the frequent bilaterality and multifocality of the pulmonary artery aneurysms make transcatheter embolization an attractive alternative to surgery in most cases. To our knowledge, few patients with Hughes-Stovin syndrome have been treated by performing embolization of a pulmonary arterial aneurysm with the use of several agents, including steel coils, Ethibloc, and isobutyl cyanoacrylate, an epoxy [4,6,7].

This is the first reported successful use of transcatheter embolization with steel coils and a vascular plug of a pulmonary artery aneurysm in a patient with Hughes-Stovin syndrome. The Amplatzer Vascular Plug is a type of nitinol-based, self-expanding device that is used mostly to occlude the internal iliac artery in patients undergoing aortoiliac or common iliac aneurysm endograft repair, or both [8]. Its use has been extended to the treatment of many patients undergoing emergency embolization and has had a high technical success rate [9].

## Conclusions

The less invasive procedure described in this report enables selective treatment of the arterial complications of Hughes-Stovin syndrome, such as a single large pulmonary artery aneurysm, and contributes to the best multidisciplinary approach to treating these patients.

## Consent

Written informed consent was obtained from the patient for publication of this case report and any accompanying images. A copy of the written consent is available for review by the Editor-in-Chief of the journal.

## Competing interests

The authors declare that they have no competing interests.

## Authors' contributions

TVD analyzed and interpreted the patient data regarding diagnosis and treatment. VG analyzed the patient data and contributed to the writing of the manuscript. TIA contributed to the writing of the manuscript. TVD interpreted the patient data and conducted patient follow-up. BEN was the main contributor to the endovascular treatment of the patient. All authors read and approved the final manuscript.
